# Phylogenomic Analysis of *Dichrocephala benthamii* and Comparative Analysis within Tribe Astereae (Asteraceae)

**DOI:** 10.1590/1678-4685-GMB-2023-0340

**Published:** 2024-10-21

**Authors:** Hui Chen, Tingyu Li, Xinyu Chen, Xinyi Zheng, Tianmeng Qu, Bo Li, Zhixi Fu

**Affiliations:** 1 Sichuan Normal University, Key Laboratory of Land Resources Evaluation and Monitoring in Southwest, Chengdu, Sichuan, China.; 2 Sichuan Normal University, College of Life Sciences, Chengdu, Sichuan, China.; 3 Sichuan Environmental Monitoring Center, Chengdu, Sichuan, China.; 4 Sichuan Normal University, Sustainable Development Research Center of Resources and Environment of Western Sichuan, Chengdu, Sichuan, China.

**Keywords:** Dichrocephala benthamii, chloroplast genome, phylogenetic analysis, comparative analysis

## Abstract

*Dichrocephala benthamii* C. B. Clarke has long been used as traditional Chinese medicine. However, the chloroplast (cp) genome of *D. benthamii* is poorly understood so far. In this study, we sequenced and analyzed the cp genome of *D. benthamii*. The results showed that the cp genome is 152,350 bp in length, with a pair of inverted repeat regions (IRa and IRb, each 24,982 bp), a large single-copy (LSC) region comprising 84,136 bp, and a small single-copy (SSC) region comprising 18,250 bp. The GC content of the cp genome was 37.3%. A total of 134 genes were identified, including 87 protein-coding genes (CDS), 38 tRNA genes, 8 rRNA genes, and 1 pseudogene (*ycf1*). Expansion or contraction of IR regions were detected in *D. benthamii* and other species of the tribe Astereae. Additionally, our analyses showed the types of sequence repeats and the highly variable regions discovered by analyzing the border regions, sequence divergence, and hot spots. The phylogenetic analysis revealed *D. benthamii* is the basal group of Astereae. The results of this study will be a significant contribution to the genetics and species identification related to *D*. *benthamii*.

The family Asteraceae contains about 1600-1700 genera and 26,000 species (Funk et al., 2005). The plants in Asteraceae are characterized by the distinctive capitula. The tribe Astereae, a second largest tribe of Asteraceae, includes about 225 genera and 3,100 species, of which 29 genera and 237 species are native to China (112 species are endemic) (Brouillet et al., 2009).The whole plant of *D. benthamii* is used medicinally as common herb among Dai nationality of China for the treatment of indigestion, common cold, fever in children, pneumonia and hepatitis (Song et al., 2017). Previous research on *D. benthamii* has focused on medicinal and pharmacological studies (Song *et al*., 2017).

The analysis of cp genomes has become a major research focus in plant evolution and systematics (Vargas et al., 2017). To date, sequences of *D. benthamii* (*rbcL*, *matK*, and *rpoC*) have been reported (https://www.ncbi.nlm.nih.gov). However, available genetic data for comparative genomic studies of *D. benthamii* and related genera are limited. In the study, we sequenced, assembled and annotated the complete cp genome of *D. benthamii*. The objectives of this study were to: 1) identify and characterize the cp genome structure and sequence differentiation throughout the plastids; and 2) assess the phylogenetic relationships among *D. benthamii* and other Asteraceae species, which may be useful for further speciation studies.

Fresh leaves of *D. benthamii* were collected from Yuexi county, Liangshan Prefecture, Sichuan Province, China (Figure S1). The voucher specimen was collected and placed in the herbarium of the Sichuan Normal University, China (SCNU) (Contact: Zhixi Fu, fuzx2017@sicnu.edu.cn) under the voucher number: Junjia Luo 311. Total genomic DNA was isolated using a modified CTAB method (Allen et al., 2006). DNA libraries were constructed using the Illumina Paired-End DNA Library Kit. The qualified library was sequenced using the Illumina NovaSeq 6000 platform with a sequencing read length was 150 bp. The cp genome of *D. benthamii* was assembled using SPAdes software (v3.15.1) (Bankevich et al., 2012). Subsequently, the results were annotated using PGA based on the reference cp genome sequence of *Eschenbachia blinii* (H.Lév.) (NC 037605.1) (Qu et al., 2019). The cp genome sequence of *D. benthamii* was uploaded to NCBI with the accession number ON751565. The complete cp genome map was constructed using OGDRAW (Greiner et al., 2019).

IRscope is a bioinformatics tool used to visualize the expansions and contractions of cp genomes (Amiryousefi et al., 2018). Additionally, as an online server, mVISTA was employed to compare DNA sequences of 6 species in tribe Astereae (Frazer et al., 2004).

Simple sequence repeats (SSRs) in the plastomes were detected using the Perl script MISA (Beier et al., 2017). The repeat units were set to 10 for mononucleotides, 5 for dinucleotides, 4 for trinucleotides and 3 for hexanucleotides, respectively.

MEGA v.7.0 was used to analyze the synonymous codon usage and the relative synonymous codon usage (RSCU) of the *D. benthamii* cp genome.

The 28 complete cp sequences were downloaded from NCBI and combined with the sequenced complete cp sequence of *D. benthamii*. *Achillea millefolium* L. and *Ajania pacifica* (Nakai) K. Bremer & Humphries (tribe Anthemideae) were included as outgroups (Table S1). Phylogenetic relationship reconstruction analysis using Maximum Likelihood (ML) method was conducted with RAxML (Stamatakis, 2014) based on the GTRGAMMA model on the CIPRES platform (Miller et al., 2010). Default settings were used for other parameters. Bootstrap analysis with 1,000 replicates was performed to assess bootstrap values (BS) for each node of the phylogenetic tree. 

The complete cp genome of *D. benthamii* was 152,350 bp in size. It exhibited the quadripartite structure consisting of two IR regions (24,982 bp each), a LSC region (84,136 bp), and a SSC region (18,250 bp) (Figure 1). The overall GC content of the *D. benthamii* cp genome was 37.3%, similar to that of the other 5 Astereae species (37.28% to 37.36%) (Table 1). The GC contents of the SSC regions, LSC region and SSC region among 6 Astereae species varied from 31.2% to 31.33%, from 35.15% to 35.3% and from 42.99% to 43.06%, respectively. The consistent GC content may play a role in maintaining genetic stability (Niu et al., 2017). *D. benthamii* possessed 134 unique genes, comprising 87 protein-coding genes, 38 tRNA genes, and 8 rRNA genes. Seven tRNA genes and all rRNA genes were located in the IR regions, contributing to the high GC content of these regions. This phenomenon has been associated with the presence of NADH (Shen et al., 2017). Fifteen genes contained one intron, while three genes possessed two introns (Table S2).

**Figure 1 -:**
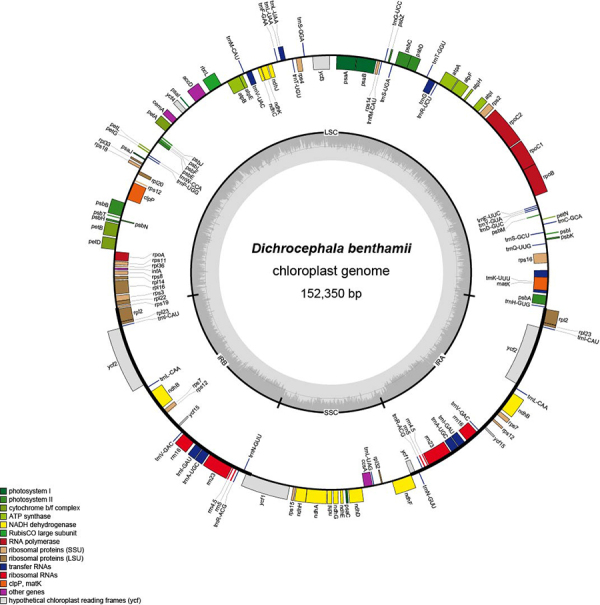
Gene map of the complete chloroplast genomes of *D. benthamii*. Annotated genes are colored according to functional categories whereby the genes outside the circle were transcribed clockwise, while the genes placed inside the circle were transcribed counterclockwise. The dark grey color in the inner circle represents GC content, whereas the light grey color corresponds to AT content.

**Table 1 -:** Summary of the complete chloroplast genomes of 6 species of Astereae.

Species	Genome Size (bp)	LSC (bp)	IR (bp)	SSC (bp)	GC Content (%)			
					All	LSC	IR	SSC
*Dichrocephala benthamii*	152,350	84,136	24,982	18,250	37.3	35.25	42.98	31.2
*Aster ageratoides*	153,071	84,896	24,953	18,269	37.28	35.15	43.06	31.33
*Aster pekinensis*	152,815	84,530	25,033	18,219	37.3	35.22	42.99	31.33
*Aster tataricus*	152,992	84,698	25,022	18,250	37.26	35.15	43.01	31.26
*Heteropappus gouldii*	152,450	84,226	25,011	18,202	37.36	35.3	43.03	31.3
*Heteropappus sericophylla*	152,214	84,369	24,983	18,293	37.32	35.23	43.04	31.32

Analysis of the IR boundaries in six Astereae species revealed varying contractions and expansions of the IR, leading to variability in genome length (Kim and Lee, 2004) (Figure S2). The *rps19* was located at the LSC-IRb border regions, with variations in sizes (17bp to 62bp). The *ycf1* spanned the SSC-IRa junction. The *rpl12* was entirely within the IR region, located 115bp away from the LSC. However, the pseudogene *ycf1* of *D. benthamii* spanned the boundary between the SSC and IRa regions (6bp). The mVISTA-based identity plot revealed high sequence similarity with a few variants. These variations were typically observed in the intergenic spacers (IGS) rather than coding-regions, suggesting that coding regions were more conserved than non-coding regions (Figure S3). Overall, more variations were observed in the LSC and SSC regions compared to the IR regions. This result is consistent with patterns observed in cp genomes of other Asteraceae species (Loeuille et al., 2021). The high variation observed in the LSC and SSC regions was primarily attributed to non-coding sequences.

In this study, 532 SSRs were identified across six species of Astereae, with their counts being very similar (ranging from 84 to 100) (Figure 2). *Aster tataricus* L.f. exhibited the highest number of SSRs (100), while *Aster ageratoides* Turcz. and *Heteropappus gouldii* (C.E.C.Fisch.) Grierson had the lowest (84). The detected SSRs encompassed six types: mononucleotides (38.16%), dinucleotides (17.86%), trinucleotides (20.49%), tetranucleotides (16.17%), pentanucleotides (6.02%), and hexanucleotides (1.32%) (Figure 2). These SSRs could be utilized to explore the genetic structure, diversity, phylogeny, and differentiation of Astereae and other *Dichrocephala* species. Additionally, 80 SSRs were identified in *D. benthamii*, comprising motifs such as A/T, AT/AT, AAT/AAT, AAAT/ATTT, AATT/AATT, AAACT/AGTTT, AATAT/ ATATT (Table S3). The preference for AT-rich motifs is consistent with findings from many plant plastids (Zavala-Paez et al., 2020).

**Figure 2 -:**
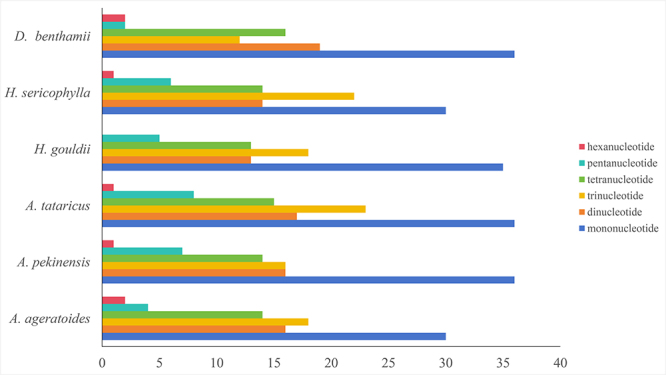
Analysis of SSRs in 6 species of Astereae plastid genomes species.

By calculating the RSCU values for all protein-coding genes in the cp genomes of *D. benthamii*, a total of 30,600 codons were identified. Thirty-one types of codons exhibited greater preference (RSCU > 1) (Figure S4). Serine showed no preferences (RSCU = 1), while the remaining codons were less preferred. Notably, no codons were extremely rare (RSCU < 0.1). Among the 20 amino acids, leucine (10.67%) constituted the largest proportion, while cysteine (1.12%) accounted for the smallest (Table S4). In other angiosperm cp genomes, leucine and cysteines have been reported the most and least abundant amino acids, respectively (Sharp and Li, 1987). Intriguingly, with the exception of UUG, all preferentially used codons were ended with A/U. This result is consistent with observations in other Astereae species (Chen et al., 2024). The high proportion of A/U is the major force of deviation (Claude and Park, 2020).

Phylogenomic analysis based on cp genome data identified several clades (Figure 3). The tribe Astereae was found to form a monophyletic group. *D. benthamii* and other Asteraceae species clustered into a clade supported by a bootstrap value of 100%. *D. benthamii* was located at the base of the phylogenetic tree, which was in agreement with Brouillet et al. (2009). This study will fill a gap in the research of the cp genome of *Dichrocephala* plants and provide a wealth of information for the taxonomic study of this genus in Asteraceae.

**Figure 3 -:**
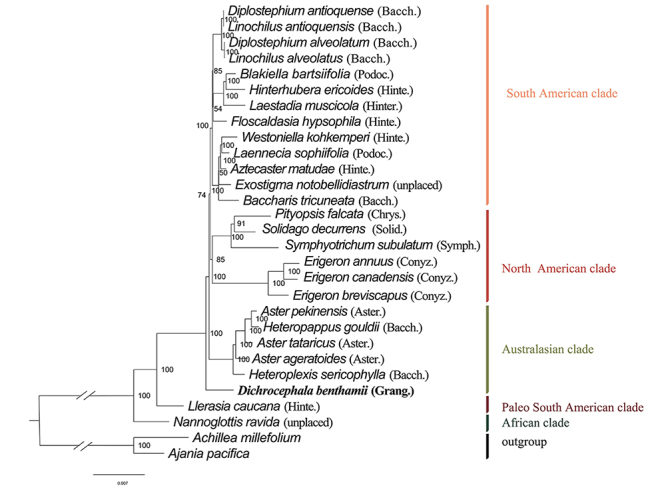
Maximum likelihood tree of *D. benthamii* reconstructed based on 29 complete cp genome sequences. The species names in bold font represent our sequenced species plastomes. (the subtribe is marked by the Brouillet et al., 2009; Bacch.: Baccharidinae; Podoc.: Podocominae; Hinter.: Hinterhuberinae; Chrys.: Chrysopsidinae; Solid.: Solidagininae; Symph.: Symphyotrichinae; Conyz.: Conyzinae; Aster.: Asterinae; Grang.: Grangeinae; pentagram: Astereae originated in Africa).

This study firstly reported the cp genomes of *D. benthamii*. Comparative analyses revealed the genome’s structure and composition, variable regions and SSR markers. Phylogenetic analysis indicated *D. benthamii* is at the base of the phylogenetic tree in Astereae. Thus, the complete cp genome of *D. benthamii* provides valuable genetic insights into this genus and establishes a foundation for investigating population evolution among Astereae species.

## Supplementary material

The following online material is available for this article:

Table S1 - information on the 28 species used in the study.

Table S2 - List of genes found in *D. benthamii.*

Table S3 - SSR in the cp genome of *D. benthamii*.

Table S4 - Codon usage in the chloroplast genomes of *D. benthamii.*

Figure S1 - The photos of *D. benthamii* were taken by Xudong, Ma without any copyright issues.

Figure S2 - Comparison for border positions of LSC, SSC, and IR regions among the 6 species of tribe Astereae.

Figure S3 - The cp genomes of 6 species of Astereae were compared using the mVista program with *D. benthamii* as the reference.

Figure S4 - Codon content of 20 amino acid and stop codons in all protein-coding genes of the CP genome of *D. benthamii*.
