# Supplementary material for: Phylogenomic Analysis of Dichrocephala benthamii and Comparative Analysis within Tribe Astereae (Asteraceae)
Source: Genet Mol Biol. 2024 Oct 21;47(4):e20230340. doi: 10.1590/1678-4685-GMB-2023-0340 (PMC11495966; doi:10.1590/1678-4685-GMB-2023-0340)
Supplement: Table S1 - [file 1415-4757-GMB-47-4-e20230340-s1.pdf]

Genetics and Molecular Biology
https://doi.org/10.1590/1678-4685-GMB-2023-0340

Supplementary Material to "Phylogenomic Analysis of *Dichrocephala benthamii* and Comparative Analysis within Tribe Astereae (Asteraceae)"

Table S1 - information on the 28 species used in the study.

Species name	GenBank accession	Tribe	Subtirbe
Ajania pacifica (Nakai) K.Bremer & Humphries	NC_050690	Anthemideae	Artemisiinae
Achillea millefolium L.	ON320384.1	Anthemideae	Matricariinae
Aster ageratoides (Nakai) K.Bremer & Humphries	NC_058273.1	Astereae	Asterinae
Aster pekinensis (Homce) F.H.Chen	MW255593.1	Astereae	Asterinae
Aster tataricus L.f.	NC_042913.1	Astereae	Asterinae
Baccharis tricuneata (L.f.) Pers	KX063888.1	Astereae	Baccharidinae
Diplostephium alveolatum Cuatrec.	NC_034847.1	Astereae	Baccharidinae
Diplostephium antioquense Cuatrec.	NC_034876.1	Astereae	Baccharidinae
Heteropappus gouldii (C. E. C. Fisch.) Grierson	NC_061598.1	Astereae	Baccharidinae
Heteroplexis sericophylla Y. L. Chen	MK942054.1	Astereae	Baccharidinae
Linochilus alveolatus (Cuatrec.) Saldivia & O.M.Vargas	KX063856.1	Astereae	Baccharidinae
Linochilus antioquensis (Cuatrec.) Saldivia & O.M.Vargas	KX063898.1	Astereae	Baccharidinae
Pityopsis falcata (Pursh) Nutt.	KY045817.1	Astereae	Chrysopsidinae
Erigeron annuus (L.) Pers.	MZ361990.1	Astereae	Conyzinae
Erigeron breviscapus (Vaniot) Hand.-Mazz.	NC_043882.1	Astereae	Conyzinae
Erigeron canadensis Ten.	MT806101.1	Astereae	Conyzinae
Dichrocephala benthamii C.B.Clarke	ON751565	Astereae	Grangeinae
Aztecaster matudae (Rzed.) G.L.Nesom	KX063935.1	Astereae	Hinterhuberinae
Floscaldasia hypsophila Cuatrec.	NC_034888.1	Astereae	Hinterhuberinae
Hinterhubera ericoides Wedd.	NC_034884.1	Astereae	Hinterhuberinae
Laestadia muscicola Wedd.	NC_034858.1	Astereae	Hinterhuberinae
Llerasia caucana (S.F.Blake) Cuatrec.	NC_034821.1	Astereae	Hinterhuberinae

Species name	GenBank accession	Tribe	Subtirbe
Westoniella kohkemperi Cuatrec.	KX063921.1	Astereae	Hinterhuberinae
Blakiella bartsiifolia (S.F.Blake) Cuatrec.	NC_034866.1	Astereae	Podocominae
Laennecia sophiifolia (Kunth) G.L.Nesom	NC_034877.1	Astereae	Podocominae
Solidago decurrens Lour.	NC_053705.1	Astereae	Solidagininae
Symphyotrichum subulatum (Michx.) G.L.Nesom	NC_050667.1	Astereae	Symphyotrichinae
Exostigma notobellidiastrum (Griseb.) G.Sancho	NC034864.1	Astereae	unplaced
Nannoglottis ravida (C.Winkl.) Y.L.Chen	NC_053322.1	Astereae	unplaced
