# Supplementary material for: Phylogenomic Analysis of Dichrocephala benthamii and Comparative Analysis within Tribe Astereae (Asteraceae)
Source: Genet Mol Biol. 2024 Oct 21;47(4):e20230340. doi: 10.1590/1678-4685-GMB-2023-0340 (PMC11495966; doi:10.1590/1678-4685-GMB-2023-0340)
Supplement: Table S2 - [file 1415-4757-GMB-47-4-e20230340-s2.pdf]

Genetics and Molecular Biology
https://doi.org/10.1590/1678-4685-GMB-2023-0340

Supplementary Material to "Phylogenomic Analysis of *Dichrocephala benthamii* and Comparative Analysis within Tribe Astereae (Asteraceae)"

Table S2 - List of genes found in *D. benthamii.*

Category of Genes	Group of Genes	Name of Genes	Number of genes
Photosynthesis	Subunits of photosystem I	*psaA*, *psaB*, *psaC*, *psaI*, *psaJ*	5
	Subunits of photosystem II	*psbA*, *psbB*, *psbC*, *psbD*, *psbE*, *psbF*, *psbH*, *psbI*, *psbJ*, *psbK*, *psbL*, *psbM*, *psbN*, *psbT*, *psbZ*	15
	Subunits of NADH dehydrogenase	*ndhA**, *ndhB**(x2), *ndhC*, *ndhD*, *ndhE*, *ndhF*, *ndhG*, *ndhH*, *ndhI*, *ndhJ*, *ndhK*	12
	Subunits of cytochrome b/f complex	*petA*, *petB**, *petD**, *petG*, *petL*, *petN*	6
	Subunits of ATP synthase	*atpA*, *atpB*, *atpE*, *atpF**, *atpH*, *atpI*	6
	Large subunit of rubisco	*rbcL*	1
Self-replication	Proteins of large ribosomal subunit	*rpl14*, *rpl16*, *rpl2**(x2), *rpl20*, *rpl22*, *rpl23*(x2), *rpl32*, *rpl33*, *rpl36*	11
	Proteins of small ribosomal subunit	*#rps19*, *rps11*, *rps12***(x2), *rps14*, *rps15*, *rps16**, *rps18*, *rps19*, *rps2*, *rps3*, *rps4*, *rps7*(x2), *rps8*	15
	Subunits of RNA polymerase	*rpoA*, *rpoB*, *rpoC1**, *rpoC2*	4

Category of Genes	Group of Genes	Name of Genes	Number of genes
	Ribosomal RNAs	*rrn16*(x2), *rrn23*(x2), *rrn4.5*(x2), *rrn5*(x2)	8
	Transfer RNAs	*trnA-UGC*(2),trnC-GCA,trnD-GUC,trnE-UUC,trnF-GAA,trnG*,trnG-UCC,trnH-GUG,trnI-CAU(2),trnI-GAU*(2),trnK-UUU*,trnL-CAA(2),trnL-UAA,trnL-UAA*,trnL-UAG,trnM-CAU,trnN-GUU(2),trnP-UGG,trnQ-UUG,trnR-ACG(2),trnR-UCU,trnS-GCU,trnS-GGA,trnS-UGA,trnT-GGU,trnT-UGU,trnV-GAC(2),trnV-UAC*,trnW-CCA,trnY-GUA,trnfM-CAU*	38
Other genes	Maturase	*matK*	1
	Protease	*clpP***	1
	Envelope membrane protein	*cemA*	1
	Acetyl-CoA carboxylase	*accD*	1
	c-type cytochrome synthesis gene	*ccsA*	1
	Translation initiation factor	*infA*	1
Genes of unknown function	Conserved hypothetical chloroplast ORF	*#ycf1, ycf1, ycf15(2), ycf2(2), ycf3**, ycf4*	8

Notes: *Gene contains one intron. **Gene contains two introns. #: Pseudo gene. (×2) indicates the number of the repeat unit is 2.
