# Supplementary material for: Phylogenomic Analysis of Dichrocephala benthamii and Comparative Analysis within Tribe Astereae (Asteraceae)
Source: Genet Mol Biol. 2024 Oct 21;47(4):e20230340. doi: 10.1590/1678-4685-GMB-2023-0340 (PMC11495966; doi:10.1590/1678-4685-GMB-2023-0340)
Supplement: Table S3 - [file 1415-4757-GMB-47-4-e20230340-s3.pdf]

Genetics and Molecular Biology
https://doi.org/10.1590/1678-4685-GMB-2023-0340

Supplementary Material to "Phylogenomic Analysis of *Dichrocephala benthamii* and Comparative Analysis within Tribe Astereae (Asteraceae)"

Table S3 - SSR in the cp genome of *D. benthamii*.

**Repeat type (number)**	**Repeat Sequence**	Number of repetitions														total
		3	4	5	6	7	8	9	10	11	12	13	14	15	16	
Mononucleotides (36)	A/T	-	-	-	-	-	-	-	19	6	5	3		1	2	36
Dinucleotides (19)	AT/AT	-	-	13	5	1										19
trinucleotide (12)	AAG/CTT	-	1	1												2
	AAT/AAT	8	2													10
Tetranucleotide (16)	AAAT/ATTT	12														12
	AATC/ATTG	1														1
	AATT/AATT	2														2
	AGAT/ATCT	1														1
Pentanucleotide (2)	AAACT/AGTTT	1														1
	AATAT/ATATT	1														1
Hexanucleotide (2)	AATCCT/AGGATT	2														2
