# Supplementary material for: Phylogenomic Analysis of Dichrocephala benthamii and Comparative Analysis within Tribe Astereae (Asteraceae)
Source: Genet Mol Biol. 2024 Oct 21;47(4):e20230340. doi: 10.1590/1678-4685-GMB-2023-0340 (PMC11495966; doi:10.1590/1678-4685-GMB-2023-0340)
Supplement: Table S4 - [file 1415-4757-GMB-47-4-e20230340-s4.pdf]

Genetics and Molecular Biology
https://doi.org/10.1590/1678-4685-GMB-2023-0340

Supplementary Material to "Phylogenomic Analysis of *Dichrocephala benthamii* and Comparative Analysis within Tribe Astereae (Asteraceae)"

Table S4 - Codon usage in the chloroplast genomes of *D. benthamii.*

Amino acid	Symbol	Codon	Count	RSCU	Amino acid	Symbol	Codon	Count	RSCU
*	Ter	UAA	48	1.7778	M	Met	AUU	1	0.0128
*	Ter	UAG	19	0.7037	M	Met	CUG	0	0
*	Ter	UGA	14	0.5185	M	Met	GUG	0	0
A	Ala	GCA	359	1.1089	M	Met	UUG	0	0
A	Ala	GCC	211	0.6517	N	Asn	AAC	239	0.443
A	Ala	GCG	147	0.4541	N	Asn	AAU	840	1.557
A	Ala	GCU	578	1.7853	P	Pro	CCA	275	1.1329
C	Cys	UGC	73	0.5703	P	Pro	CCC	184	0.758
C	Cys	UGU	183	1.4297	P	Pro	CCG	142	0.585
D	Asp	GAC	180	0.3987	P	Pro	CCU	370	1.5242
D	Asp	GAU	723	1.6013	Q	Gln	CAA	648	1.5521
E	Glu	GAA	881	1.5073	Q	Gln	CAG	187	0.4479
E	Glu	GAG	288	0.4927	R	Arg	AGA	422	1.8522
F	Phe	UUC	421	0.6502	R	Arg	AGG	140	0.6145
F	Phe	UUU	874	1.3498	R	Arg	CGA	303	1.3299
G	Gly	GGA	599	1.5041	R	Arg	CGC	89	0.3906
G	Gly	GGC	188	0.4721	R	Arg	CGG	98	0.4301
G	Gly	GGG	286	0.7181	R	Arg	CGU	315	1.3826
G	Gly	GGU	520	1.3057	S	Ser	AGC	98	0.3488
H	His	CAC	135	0.5153	S	Ser	AGU	354	1.2598
H	His	CAU	389	1.4847	S	Ser	UCA	342	1.2171
I	Ile	AUA	607	0.9435	S	Ser	UCC	246	0.8754
I	Ile	AUC	358	0.5565	S	Ser	UCG	135	0.4804
I	Ile	AUU	965	1.5	S	Ser	UCU	511	1.8185

**Amino acid**	**Symbol**	**Codon**	**Count**	**RSCU**	**Amino acid**	**Symbol**	**Codon**	**Count**	**RSCU**
K	Lys	AAA	895	1.4992	T	Thr	ACA	356	1.2255
K	Lys	AAG	299	0.5008	T	Thr	ACC	221	0.7608
L	Leu	CUA	316	0.778	T	Thr	ACG	115	0.3959
L	Leu	CUC	155	0.3816	T	Thr	ACU	470	1.6179
L	Leu	CUG	150	0.3693	V	Val	GUA	479	1.5218
L	Leu	CUU	524	1.2901	V	Val	GUC	154	0.4893
L	Leu	UUA	793	1.9524	V	Val	GUG	166	0.5274
L	Leu	UUG	499	1.2286	V	Val	GUU	460	1.4615
M	Met	AUA	0	0	W	Trp	UGG	403	1
M	Met	AUC	1	0.0128	Y	Tyr	UAC	152	0.3543
M	Met	AUG	547	6.9745	Y	Tyr	UAU	706	1.6457
