# Supplementary material for: Phylogenomic Analysis of Dichrocephala benthamii and Comparative Analysis within Tribe Astereae (Asteraceae)
Source: Genet Mol Biol. 2024 Oct 21;47(4):e20230340. doi: 10.1590/1678-4685-GMB-2023-0340 (PMC11495966; doi:10.1590/1678-4685-GMB-2023-0340)
Supplement: Figure S1 - [file 1415-4757-GMB-47-4-e20230340-s5.pdf]

Genetics and Molecular Biology
https://doi.org/10.1590/1678-4685-GMB-2023-0340

Supplementary Material to “Phylogenomic Analysis of *Dichrocephala benthamii* and Comparative Analysis within Tribe Astereae (Asteraceae)”

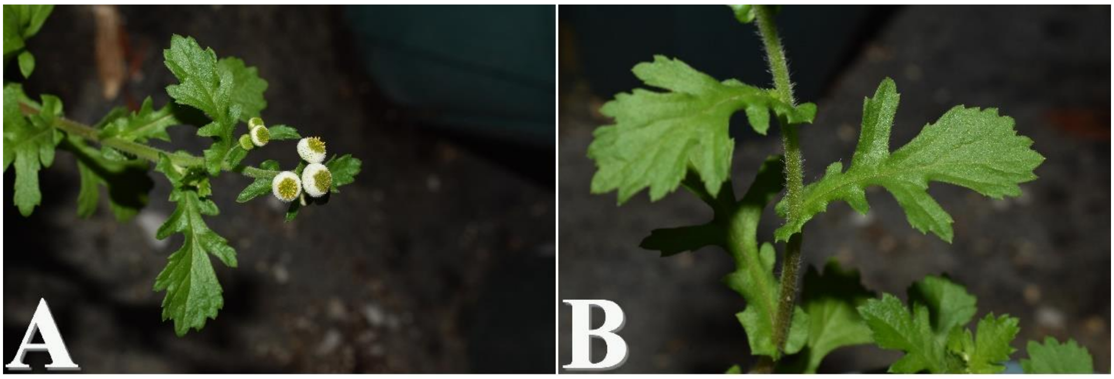

Figure S1 - The photos of *D. benthamii* were taken by Xudong, Ma without any copyright issues. Panel A shows its flowers and Panel B shows its leaves. The plant's coordinate is 28.639801 N, 102.507680 E. It's an annual herb 6.5-35 cm tall. Leaves sessile, blade ovate. Capitula globose, 3-5 mm in diam, bloom in year-round. Achenes straw-colored, obovoid. This plant thrives in grasslands in valleys, riverbanks, fields, wastelands, roadsides at an altitude of 700-3200 metres.
