# Supplementary material for: Phylogenomic Analysis of Dichrocephala benthamii and Comparative Analysis within Tribe Astereae (Asteraceae)
Source: Genet Mol Biol. 2024 Oct 21;47(4):e20230340. doi: 10.1590/1678-4685-GMB-2023-0340 (PMC11495966; doi:10.1590/1678-4685-GMB-2023-0340)
Supplement: Figure S2 - [file 1415-4757-GMB-47-4-e20230340-s6.pdf]

Genetics and Molecular Biology
https://doi.org/10.1590/1678-4685-GMB-2023-0340

Supplementary Material to “Phylogenomic Analysis of *Dichrocephala benthamii* and Comparative Analysis within Tribe Astereae (Asteraceae)”

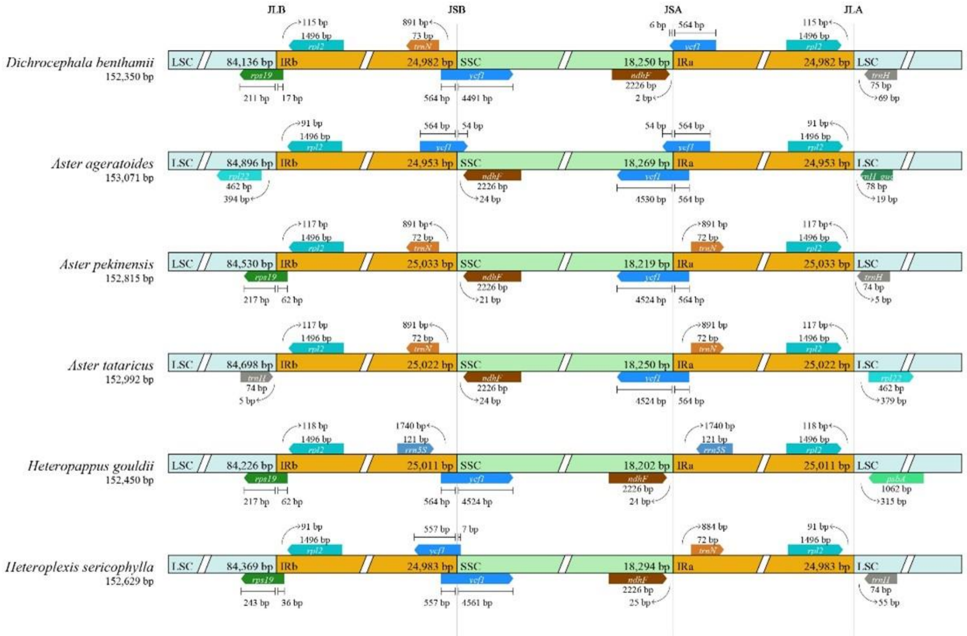

Figure S2 - Comparison for border positions of LSC, SSC, and IR regions among the 6 species of tribe Astereae. The boxes denote genes, and the gap between the genes and the boundaries is indicated by the number of bases unless the gene coincides with the boundary. Extensions of genes are shown above the boxes.
