# Supplementary material for: Phylogenomic Analysis of Dichrocephala benthamii and Comparative Analysis within Tribe Astereae (Asteraceae)
Source: Genet Mol Biol. 2024 Oct 21;47(4):e20230340. doi: 10.1590/1678-4685-GMB-2023-0340 (PMC11495966; doi:10.1590/1678-4685-GMB-2023-0340)
Supplement: Figure S3 - [file 1415-4757-GMB-47-4-e20230340-s7.pdf]

Genetics and Molecular Biology
https://doi.org/10.1590/1678-4685-GMB-2023-0340

Supplementary Material to "Phylogenomic Analysis of *Dichrocephala benthamii* and Comparative Analysis within Tribe Astereae (Asteraceae)"

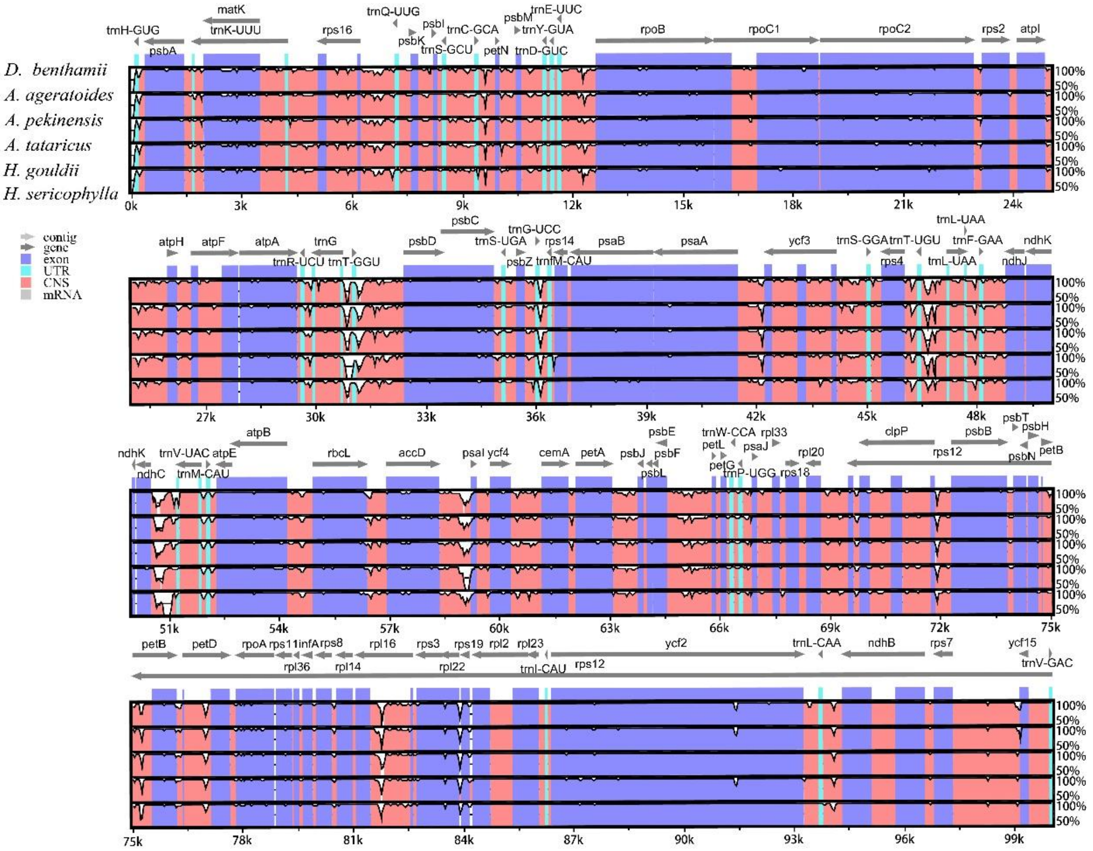

Figure S3 - The cp genomes of 6 species of Astereae were compared using the mVista program with *D. benthamii* as the reference. The arrow indicates the position and orientation of the gene. The Y -axis represents the percentage of identity among genome sequences (50%–100%).
